# Multi-omics elucidates the regulatory mechanisms of tryptophan in gut health of weaned piglets

**DOI:** 10.1186/s42523-026-00586-1

**Published:** 2026-06-03

**Authors:** Xiaohong Hou, Yawei Fu, Zengqiang Jia, Lingrui Hou, Yulong Yin, Kang Xu

**Affiliations:** 1 College of Veterinary Medicine, Henan Agricultural University, Zhengzhou, 450000 China; 2 Hunan Provincial Key Laboratory of Animal Nutritional Physiology and Metabolic Process, National Engineering Laboratory for Pollution Control and Waste Utilization in Livestock and Poultry Production, Institute of Subtropical Agriculture, Chinese Academy of Sciences, Changsha, 410125 China; 3 College of Animal Science and Technology, Hunan Agricultural University, Changsha, 410128 China

**Keywords:** Gut health, Immunomodulation, Multi-omics, Tryptophan, Weaned piglets

## Abstract

Tryptophan (Trp), an essential amino acid (AA) implicated in diverse physiological and pathological processes, remains incompletely characterized in its mechanisms regulating intestinal health in weaned piglets. In this study, 27 weaned Bama miniature pigs with highly homogeneous genetic characteristics (6.200 ± 0.242 kg) were randomly divided into three groups and fed a basal diet, a diet supplemented with 0.5-fold Trp, or a diet supplemented with 1.5-fold Trp for 21 days. We used multi-omics approaches to investigate the mechanisms by which Trp regulates intestinal health through dietary interventions with different concentrations. Both Trp-supplemented groups exhibited significantly reduced diarrhea incidence (*P* = 0.012) and improved intestinal morphology compared to the control group (*P < 0.05*). While Trp-targeted metabolomics showed no statistically significant alterations, metagenomic analysis revealed Trp-driven microbial remodeling, characterized by increased α-diversity, elevated abundances of *Deferribacteres*, *Turicibacter*, *Clostridials*_*Bacteria*, and *Turicibacter*_*Sanguinis*, alongside decreased *Tenericutes* and *Chryseobacterium*. Transcriptome analysis further identified immune-related pathways as central targets of Trp action. Subsequent cytokine quantification confirmed Trp’s immunomodulatory effects: pro-inflammatory cytokines (IL-1β, IL-6, IL-17) decreased, while anti-inflammatory IL-10 increased. Collectively, our findings demonstrate that Trp alleviates weaning-associated intestinal dysfunction by reshaping microbial ecosystems and regulating immune homeostasis.

## Introduction

Early weaning has become a basic practice in contemporary swine production systems to optimize the reproductive potential of sows [1]. During this critical developmental phase, piglets have not yet formed a mature digestive system and immune system. Compounded by abrupt environmental and dietary changes, this process readily induces weaning stress in piglets, leading to intestinal inflammation, intestinal atrophy, and reduced nutrient absorption. This severely affects their growth performance, metabolism, and immune functions, while simultaneously triggering intestinal microbiota dysbiosis [2, 3]. Exploring green solutions based on nutritional modulation, especially improving intestinal health through precise modulation of dietary composition, has become a hot research priority.

Tryptophan (Trp), the only essential amino acid (AA) containing an indole ring, has garnered significant attention due to its unique metabolic pathway and broad physiological functions [4, 5]. As a multifunctional feed additive, Trp serves primarily to increase feed intake [6], alleviate transport stress [7], regulate metabolism [8] and improve intestinal health [9]. Experimental evidence demonstrates that Trp administration mitigates chronic unpredictable mild stress-induced intestinal barrier impairment, attenuates colonic inflammatory responses, and ameliorates anxiety-related behavioral phenotypes [10]. Mechanistically, Trp exerts antioxidative and anti-inflammatory effects via targeted modulation of the 5′-adenosine monophosphate-activated protein kinase and nuclear factor κB (NF-κB) pathways [11]. Notably, adequate dietary Trp protects piglets from pathogenic bacteria by promoting the secretion of β-defensin from the intestinal mucosa of weaned piglets [12]. Additionally, Trp can be metabolized by gut microbes into active substances such as indole, which participates in important physiological regulatory processes [13]. It is worth noting that indole derivatives can serve as endogenous ligands for the aryl hydrocarbon receptor (AHR), a transcription factor widely expressed in immune cell lineages [14]. Activation of the AHR orchestrates multifaceted responses, including maintaining intestinal barrier homeostasis [15], and initiating immunomodulatory signaling [16]. Furthermore, Trp supplementation during growing-finishing phases reduces intestinal permeability by upregulating zonula occludens-1 expression and improving tight junction protein functionality [17]. These findings collectively underscore the dual role of Trp as a nutrient and signaling molecule in orchestrating gut health.

Maintaining intestinal health in weaned piglets is critical for nutrient absorption, immune defense and microbial balance. However, the mechanistic interplay between microbial communities and host physiology in Trp metabolism remains unresolved. To investigate the aforementioned mechanisms on a stable metabolic baseline with minimal genetic background noise, this study employed Bama miniature pigs, a model characterized by a consistent genetic background and moderate growth with stable metabolism [18]. Therefore, we employed Trp targeted metabolomics, metagenomics and transcriptomics to explore the effects of different concentrations of Trp on intestinal health in weaned piglets, thereby providing a theoretical framework for optimizing Trp-mediated regulation of intestinal immune functions in swine nutrition.

## Materials and methods

### Animal experiment design and sample collection

The animal experiment was reviewed and approved by the Animal Care and Use Committee of the Institute of Subtropical Agriculture (ISA-2020-029). All procedures were performed in accordance with the Guide for the Care and Use of Laboratory Animals.

A total of twenty-seven 30-day-old healthy weaned Bama miniature pigs (body weight 6.200 ± 0.242 kg, 13 females and 14 males) were randomly allocated to three groups (*n* = 9 per group): the CON group (basal diet without additional tryptophan supplementation), the 0.5Trp group (basal diet supplemented with 0.5-fold Trp), and the 1.5Trp group (basal diet supplemented with 1.5-fold Trp). The supplementation levels were determined based on the measured Trp content in the basal diet (0.169%). The feeding trial lasted for 21 days, and the dietary composition is presented in Table 1. All diets were formulated to meet the nutritional requirements and physiological needs of miniature pigs at this growth stage. Piglets were individually housed in the same environmentally controlled facility with ad libitum access to feed and water. The ambient temperature was maintained at 24–26 °C with a relative humidity of 40–60%. The lighting regimen was artificially controlled on a 12-h light/12-h dark cycle. The number of new cases of diarrhea piglets was recorded daily. The diarrhea rate (%) was calculated as: total number of piglets with diarrhea / (number of pigs × number of experimental days) × 100% [19].

Body weight was measured at the beginning and end of the experiment. During the experiment, the feed intake and the occurrence of diarrhea were recorded. The average daily feed intake (ADFI), average daily gain (ADG), feed-to-weight ratio (F/G), and diarrhea rate were calculated. At the end of the experiment, nine piglets in each group were euthanized with 3% pentobarbital sodium at a dose of 90 mg/kg. Then the ileum was separated by opening the abdominal cavity, quickly transferred to a centrifuge tube containing 4% paraformaldehyde for fixation, and molecular samples and contents were collected for subsequent testing. We used the RAND function in Microsoft Excel to generate random numbers for all individuals within the same group, and selected the top-ranked individuals as subsamples based on the sorted random numbers.

**Table 1:** Composition and nutrient levels (air-dry basis, %) ^a^

Items	(%)
Corn	61.00
Soybean meal	22.00
Wheat bran	14.00
Lysine	0.10
Calcium hydrogen phosphate	0.70
Talcum powder	0.90
Salt	0.30
Premix^b^	1.00
Chemical composition ^c^	
Digestibe energy, MJ/kg	13.60
Crude protein	16.06
Calcium	0.61
Total phosphorus	0.55
Arginine	0.935
Histidine	0.401
Leucine	1.240
Isoleucine	0.569
Lysine	0.778
Sulphur-containing amino acids	0.531
Phenylalanine	0.692
Threonine	0.507
Valine	0.620
Alanine	0.805
Asparagine	1.496
Glutamine	2.874
Glycine	0.639
Proline	0.927
Serine	0.764
Tyrosine	0.473
Tryptophan	0.169

^a^ Basal diet formulated according to the Chinese National Feeding Standard for Swine

^b^ Premix per kilogram of full-price diet provided: multidimensional 400 IU, choline 1600 mg, copper 19.8 mg, manganese 10.2 mg, zinc 359 mg, iron 400 mg, iodine 20 mg, selenium 0.56 mg, vitamin B1 2 mg, vitamin B2 15 mg, vitamin B12 30 μg, vitamin A 5400 IU, vitamin D3 110 IU, vitamin E 18 IU

c With the exception of Trp content, which was experimentally determined, all other nutritional components were theoretically calculated values

### Intestinal morphology

Ileal tissue samples were collected from the mid‑ileum at a location approximately 10 cm proximal to the ileocecal junction. The intestinal lumen was gently rinsed with ice‑cold phosphate‑buffered saline to remove contents, and the cleaned tissue was immediately immersed in 4% paraformaldehyde for fixation. Subsequently, three randomly selected ileal samples per group underwent standard histological processing (dewaxing, rehydration, hematoxylin and eosin staining, dehydration) for morphological evaluation. Villus height (VH) and crypt depth (CD) were measured at five randomly selected fields per section using CaseViewer software (v 2.4.0, 3DHISTECH Ltd., Hungary), and the VH / CD ratio was subsequently calculated.

### Intestinal cytokines

The concentrations of ileal cytokines interleukin (IL)-1β (Cat. No. MM-042201), IL-2 (Cat. No. MM-042101), IL-4 (Cat. No. MM-041901), IL-6 (Cat. No. MM-041801), IL-10 (Cat. No. MM-042501), IL-17 (Cat. No. MM-037701), interferon (IFN)-γ (Cat. No. MM-041201), and tumor necrosis factor (TNF)-α (Cat. No. MM-038301) of each piglet were quantified using commercial enzyme-linked immunosorbent assays (ELISA) kits (Meimian, China) following manufacturer protocols.

### Metabolomics

A proper amount of ileal contents was weighed from each piglet and homogenized with 80% methanol aqueous solution, followed by the addition of 10% methanol aqueous solution, then the mixture was centrifuged and filtered. Subsequently, the filtrate was mixed with 50% methanol and the internal standard, then analyzed via UPLC-MS/MS (ACQUITY HSS T3 column, 2.1 × 100 mm, 1.8 μm). Mobile phase: (A) 0.1% formic acid/water and (B) 0.1% formic acid/methanol with gradient elution. Mass spectrometric detection used a SCIEX AB5000 system in positive multiple reaction monitoring mode (ion source: 500 °C, 5500 V; gas pressures: curtain 30 psi, collision 6 psi, nebulizer/auxiliary 50 psi).

### Metagenome sequencing

Total genomic DNA was extracted from the ileum contents of each piglet using the TruSeq Nano DNA LT Sample Preparation Kit (Illumina, USA, Cat. No. FC-121-4001). DNA concentration and integrity were analyzed using a NanoDrop 2000 spectrophotometer (Thermo Fisher Scientific, USA) and agarose gel electrophoresis. The DNA was fragmented using an S220 Focused-ultrasonicator (Covaris, USA) and purified with Agencourt AMPure XP beads (Beckman Coulter Co., USA). Library construction was then performed using the TruSeq Nano DNA LT Sample Preparation Kit (Illumina, USA). The prepared libraries were sequenced on an Illumina NovaSeq 6000 platform using a PE150 strategy, with a sequencing depth of approximately 6 Gb per sample, generating 150-bp paired-end reads.

Raw sequencing data were first assessed for quality using FastQC, requiring a Q30 base percentage greater than 95%. Subsequently, Trimmomatic (v 0.36) was used for data processing, including adapter removal and low-quality base filtering to obtain high-quality clean reads. Host-derived contamination was removed by aligning the clean reads to the host genome using Bowtie2 (v 2.2.9). The resulting valid reads were then assembled de novo using MEGAHIT (v 1.1.2). Open reading frames (ORFs) were predicted from the assembled scaffolds using Prodigal (v 2.6.3) and translated into amino acid sequences. A non-redundant gene catalog was constructed from the predicted genes of all samples using CD-HIT (v 4.5.7), with clustering parameters set at 95% identity and 90% coverage. The longest sequence within each cluster was selected as the representative sequence to form the final non-redundant gene set. Finally, clean reads from each sample were aligned to this non-redundant gene set using Bowtie2 (v 2.2.9, parameter: 95% identity) to quantify gene abundance in each sample. Library construction, sequencing, and data analysis were performed by Shanghai OE Biotech Co., Ltd.

### Transcriptome

Three piglets from each group were randomly selected for transcriptome analysis. RNA concentration and purity were determined using a NanoDrop 2000 spectrophotometer (Thermo Fisher Scientific, USA). RNA integrity was evaluated with the RNA Nano 6000 Assay Kit on an Agilent Bioanalyzer 2100 system (Agilent Technologies, USA), and samples with RNA integrity number values above 7 were used for transcriptome sequencing. For library construction, 1 µg of total RNA per sample served as the input. Sequencing libraries were prepared using the NEBNext Ultra RNA Library Prep Kit for Illumina (New England Biolabs, USA) according to the manufacturer’s protocol. Library fragments were purified with the AMPure XP system (Beckman Coulter, USA). Then, 3 µL of USER Enzyme (New England Biolabs) was added to the size‑selected and adapter‑ligated cDNA, followed by incubation at 37 °C for 15 min and at 95 °C for 5 min before PCR amplification. Finally, PCR products were purified with the AMPure XP system, and library quality was assessed on an Agilent Bioanalyzer 2100 system (Agilent Technologies, USA).

Cluster generation was performed on a cBot Cluster Generation System using the TruSeq PE Cluster Kit v4-cBot-HS (Illumina) according to the manufacturer’s instructions. After cluster generation, the libraries were sequenced on an Illumina platform to generate paired-end reads, with an average sequencing depth of approximately 6 Gb per sample. Raw reads were processed using the BMKCloud online platform (www.biocloud.net). Briefly, adapter sequences and low‑quality reads were removed to obtain clean data. The clean reads were aligned to the pig reference genome using HISAT2 software. Differential expression analysis between groups was performed using DESeq2, and the resulting P‑values were adjusted for multiple testing using the Benjamini-Hochberg method to control the false discovery rate (FDR). Differentially expressed genes (DEGs) were identified via edgeR with thresholds of |log₂ fold change| ≥ 1 and adjusted *P*‑value < 0.05. Gene Ontology (GO) enrichment analysis of the DEGs was performed using the GOseq R package based on the Wallenius non‑central hypergeometric distribution. KEGG pathway enrichment analysis was conducted using KOBAS software.

### Real-time quantitative PCR

To validate the RNA sequencing data, six immune-related DEGs were randomly selected for confirmation by real-time quantitative PCR (RT-qPCR). Gene-specific primers were designed using Premier 5.0 (PREMIER Biosoft, USA), with β-actin as the endogenous control (Table 2). Total RNA was extracted from tissue samples using the RNAex Pro reagent (Accurate Biotechnology, Hunan, China, Cat. No. AG21101). Subsequently, cDNA was synthesized using the Evo M-MLV RT Mix Kit (Accurate Biotechnology, China, Cat. No. AG11728), and RT-qPCR was performed with SYBR Green Pro Taq HS premix (Accurate Biotechnology, China, Cat. No. AG11701) on a LightCycler 480 II real-time PCR system (Roche Diagnostics, Switzerland). All reactions were carried out in three technical replicates, and the relative gene expression levels were calculated using the 2^−ΔΔCT^ method [20].

**Table 2:** Primer sequences used in this study

Gene	Sequences	Reference
LBP	F: CCTGAGGAACACAGCCGAAT	NM_001128435.1
	R: TACAGTCTGGCTAACCGGGG	
TNFAIP3	F: CTGCCAGTTTTGTCCGCAAT	NM_001267890.1
	R: GTTCCCGTCCCCATTCGTT	
G6PC	F: GAAAGCCAAGCGAAGGTGTG	NM_001113445.1
	R: ACATGCTGGAGTTGAGAGCC	
SPP1	F: GCAGTGATAGCCTTCTGCCT	NM_214023.1
	R: TGTGGCGCTAGGAAAGTCTG	
	R: TGGGAAGGAAAGTGAAGGGC	
SERPINE1	F: AGGACCCCTGCCAGGATTAT	NM_213910.1
	R: TGCTAGGTCATGTAGCCCCT	
HTR4	F: TGATGAGCGCTACCGAAGAC	NM_001001267.1
	R: CACACTCCACTGCATCCCTT	
	R: TAGGGGGCGCCACTATCTCT	
β-actin	F: GCGTAGCATTTGCTGCATGA	XM_003357928.4
	R: GCGTGTGTGTAACTAGGGGT	

### Multi-omics integration analysis

This study employed two strategies for multi-omics data integration, both performed using R software (v 4.5.2) and its package mixOmics (v 6.18.1). To explore the overall association patterns between the microbial community and host metabolites, an unsupervised sparse partial least squares method was used for integration analysis. The relative abundance data of microbial species were subjected to centered log-ratio transformation. Metabolite abundance data were normalized by Z-score. For the multi-omics discriminant analysis of the microbiome, transcriptome, and phenotype, a supervised multi-omics sparse partial least squares discriminant analysis method was employed. Microbial data were processed using centered log-ratio transformation; transcriptomic data from differentially expressed genes were log₂-transformed followed by Z-score normalization; phenotypic data such as cytokines were directly normalized by Z-score.

### Statistical analysis

Data are presented as mean ± SEM and analyzed using one-way ANOVA in SPSS (v 25.0, IBM, USA) (significance threshold: *P* < 0.05). Tukey’s test was used to compare differences among the three groups. All statistical graphs were generated using GraphPad Prism software (v 8.0.1; GraphPad Software, USA).

## Results

### Tryptophan modulates growth performance and reduces diarrhea in weaned piglets

As shown in Table 3, at the end of the feeding trial, there was no significant difference in ADFI, ADG and F / G between the experimental group and CON group. Additionally, the CON group exhibited a significantly higher diarrhea rate compared to the experimental group (*P* = 0.012).

**Table 3:** Effect of dietary tryptophan level on growth performance of weaned piglets

Groups	CON	0.5Trp	1.5Trp	SEM	*P*
Initial weight (kg)	6.206	6.201	6.194	0.242	1.000
Final weight (kg)	9.064	10.163	10.200	0.329	0.287
ADG (kg)	0.165	0.189	0.191	0.008	0.389
ADFI (g)	439.211	441.092	462.273	9.166	0.427
F/G	2.859	2.418	2.516	0.127	0.376
Diarrhea rate (%)	2.116^a^	1.058^b^	1.058^b^	0.176	0.012

### Tryptophan modulates ileal morphology in weaned piglets

**Fig. 1:**
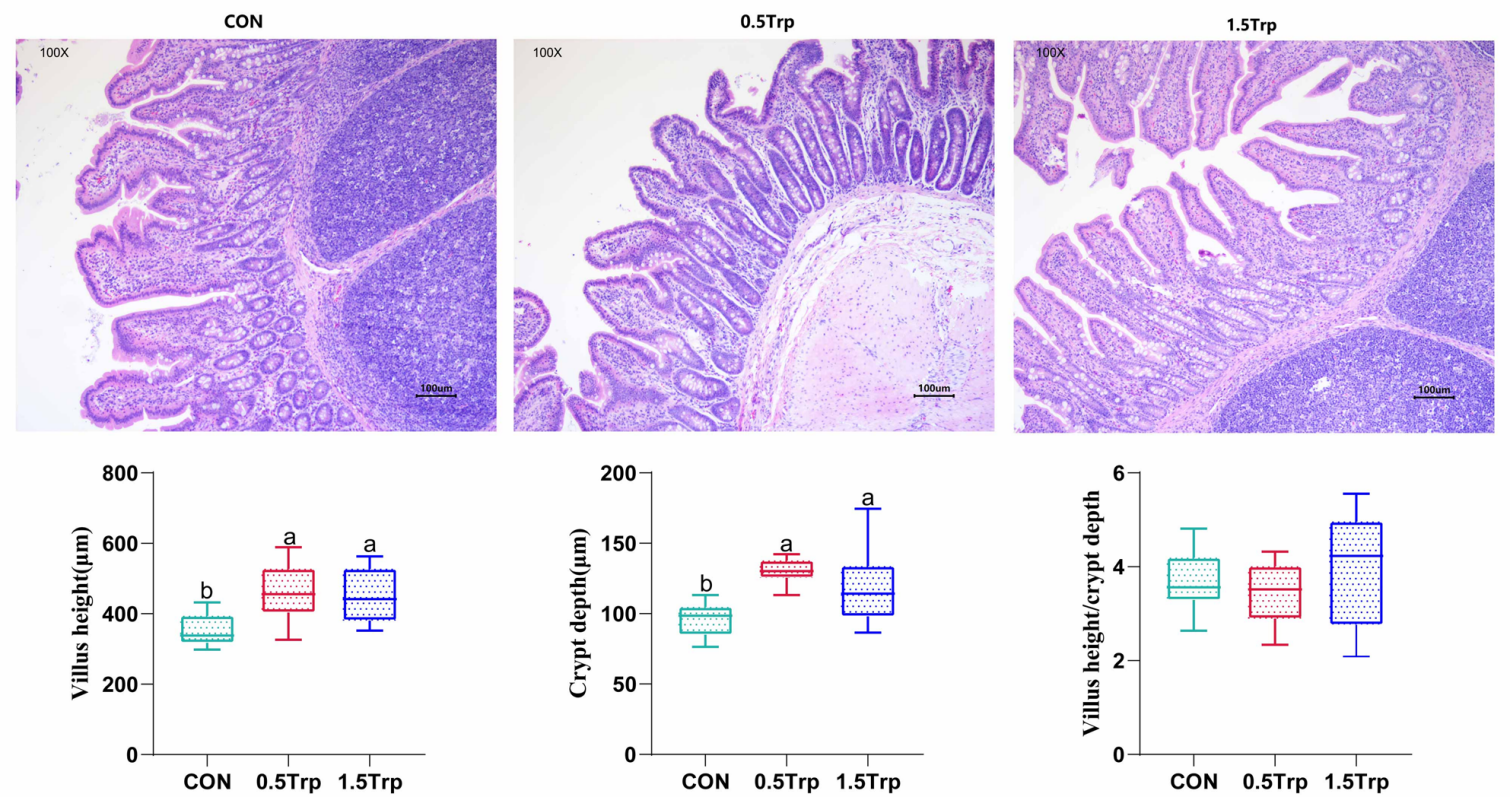
The effect of tryptophan on the morphology of the ileum Different letters represent significant differences in results (*P* < 0.05)

Compared with the CON group, the VH and CD of the ileum of piglets in the 0.5Trp group and the 1.5Trp group were significantly increased (*P* < 0.05). However, no significant differences were observed in the VH / CD ratio among the groups (Fig. 1).

### Tryptophan metabolites remained stable in the ileum of piglets

To further investigate the dynamic changes of tryptophan metabolism in piglets, we performed targeted metabolomics analysis on the ileal content. The linear equation of the standard curve and the limit of quantification are provided in Table 4. As shown in Table 5, we quantified the concentrations of various metabolites in the tryptophan metabolic pathway, including nicotinamide (NAm), serotonin (5-HT), tryptophol (IEt), kynurenic acid (KYNA), indole-3-lactic acid (ILA), indole-3-carboxaldehyde (IAld), indole-3-acetic acid (IAA), kynurenine (KYN), and tryptamine (TRM). In all comparisons, the differences in the concentrations of these metabolites among the 0.5Trp group, the 1.5Trp group, and the control group did not reach statistical significance (*P* > 0.05).

**Table 4:** Linear equation and quantitative limit of standard sample

Items	Linearity Formula	*R*	Linear ranges (ng/mL)	limit of quantification (ng/mL)
NAm	y = 0.221x-0.00642	0.9932	0.061-125	0.061
NA	y = 0.131x + 0.00762	0.9955	0.244-500	0.244
IAId	y = 0.586x-0.000813	0.9951	0.012-25	0.012
TRM	y = 0.465x + 0.00324	0.9970	0.024-50	0.024
IEt	y = 0.204x + 0.00281	0.9979	0.049-100	0.049
IAA	y = 0.0543x + 0.000906	0.9962	0.061-125	0.061
5-HT	y = 0.156x + 0.00058	0.9972	0.024-50	0.024
KYNA	y = 0.158x + 0.00185	0.9945	0.024-50	0.024
ILA	y = 0.0311x + 0.00196	0.9989	0.122-250	0.122
KYN	y = 0.0593x + 0.000434	0.9978	0.061-125	0.061

**Table 5:** Content of tryptophan metabolites in the ileum (µg/g)

Items	CON	0.5Trp	1.5Trp	SEM	*P*
NAm	1.988	1.443	2.235	0.374	0.675
NA	1.758	0.981	1.274	0.177	0.192
IAId	0.026	0.024	0.026	0.002	0.835
TRM	0.012	0.019	0.045	0.007	0.228
IEt	0.016	0.015	0.023	0.003	0.582
IAA	0.328	0.322	0.282	0.033	0.845
5-HT	0.034	0.032	0.035	0.006	0.984
KYNA	0.213	0.159	0.194	0.019	0.514
ILA	0.199	0.194	0.117	0.040	0.671
KYN	0.071	0.084	0.071	0.005	0.563

### Tryptophan significantly remodels the ileal microbiota of weaned piglets

**Fig. 2:**
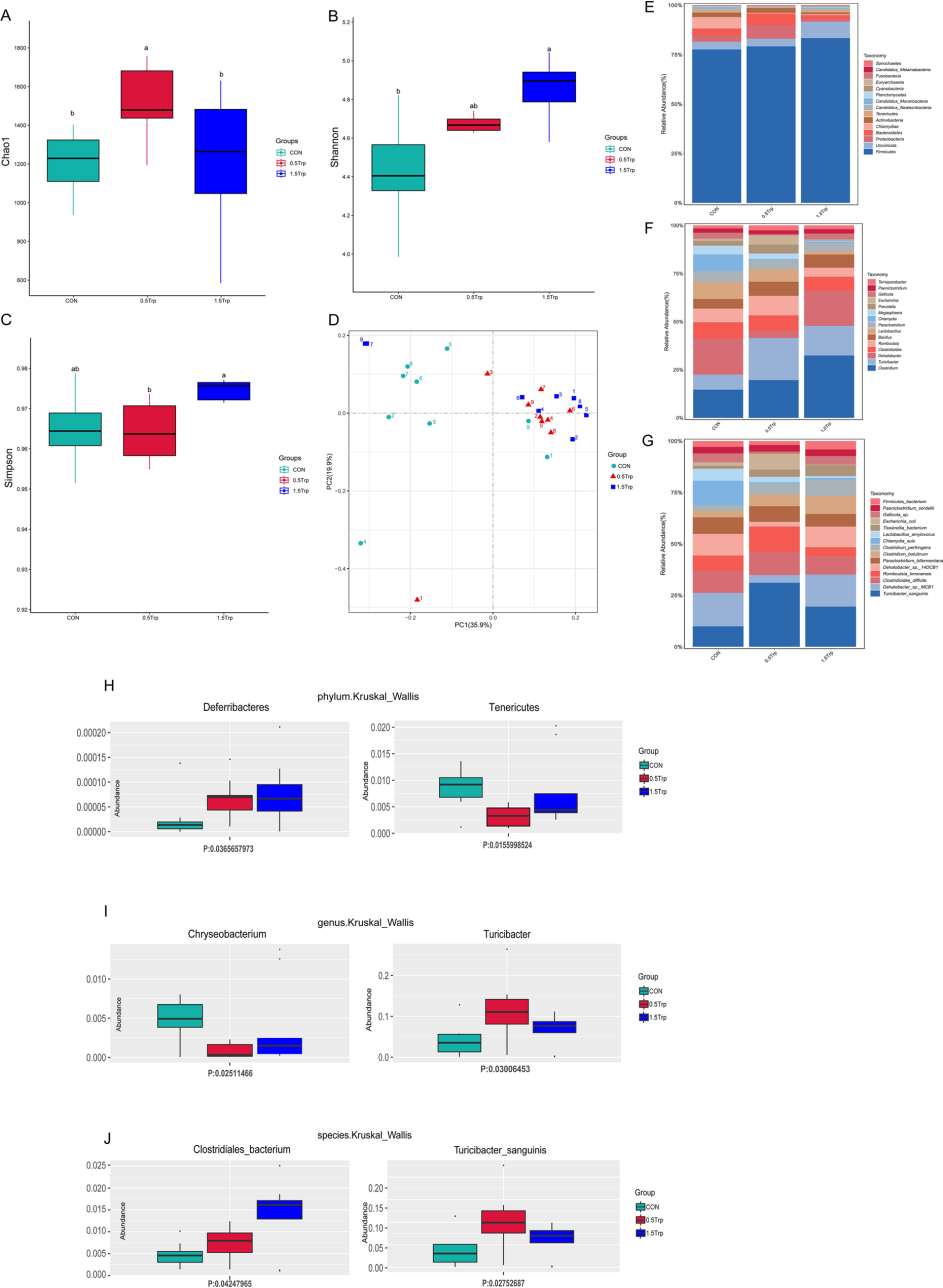
Effect of tryptophan on the diversity and composition of ileal microbiota (**A**, **B**, **C**) α-diversity Simpson (**D**) β-diversity (**E**) Species composition and relative abundance at the phylum level. (**F**) Species composition and relative abundance at the genus level. (**G**) Species composition and relative abundance at the species level. (**H**, **I**, **J**) Key species at the phylum level, genus level, and species level. Different letters represent significant differences in results (*P* < 0.05)

We calculated α-diversity using the Chao1 (Fig. 2A), Shannon (Fig. 2B) and Simpson (Fig. 2C) indices to evaluate species diversity. Principal coordinates analysis was employed to analyze β-diversity differences between groups (Fig. 2D). The results revealed that Trp supplementation significantly enhanced ileal microbiota diversity in weaned piglets. The baseline composition of the ileal microbiota was analyzed at the phylum, genus, and species levels. At the phylum level, *Firmicutes*, *Uroviricota*, *Proteobacteria*, *Bacteroidetes*, and *Chlamydiae* were the dominant microbiota (Fig. 2E). The genus-level profile was primarily composed of *Clostridium*, *Turicibacter*, *Dehalobacter*, *Clostridioides*, and *Romboutsia* (Fig. 2F); at the species level, *Turicibacter*_*sanguinis*, *Dehalobacter*_sp._MCB1, *Clostridioides*_*difficile*, *Romboutsia*_*timonensis*, and *Dehalobacter*_sp._14DCB1 were the dominant species (Fig. 2G). Given the complexity of gut ecosystem interactions, the precise mechanisms underlying Trp-mediated microbiota modulation remain unclear. To elucidate this relationship, we analyzed the abundance data of different groups using the Kruskal-Wallis method and identified significantly different groups such as *Deferribacteres*, *Tenericutes*, *Chryseobacterium*, *Turicibacter*, *Clostridiales*_*bacterium* and *Turicibacter*_*sanguinis* based on the *P*-value (Fig.  2H-J). Notably, the relative abundances of *Deferribacteres* and *Clostridiales*_*bacterium* increased with escalating Trp concentrations. *Turicibacter* and *Turicibacter*_*sanguinis* exhibited the most pronounced abundance elevation in the 0.5Trp group, whereas *Tenericutes* and *Chryseobacterium* showed marked reductions in relative abundance within the 0.5Trp treatment group.

**Fig. 3:**
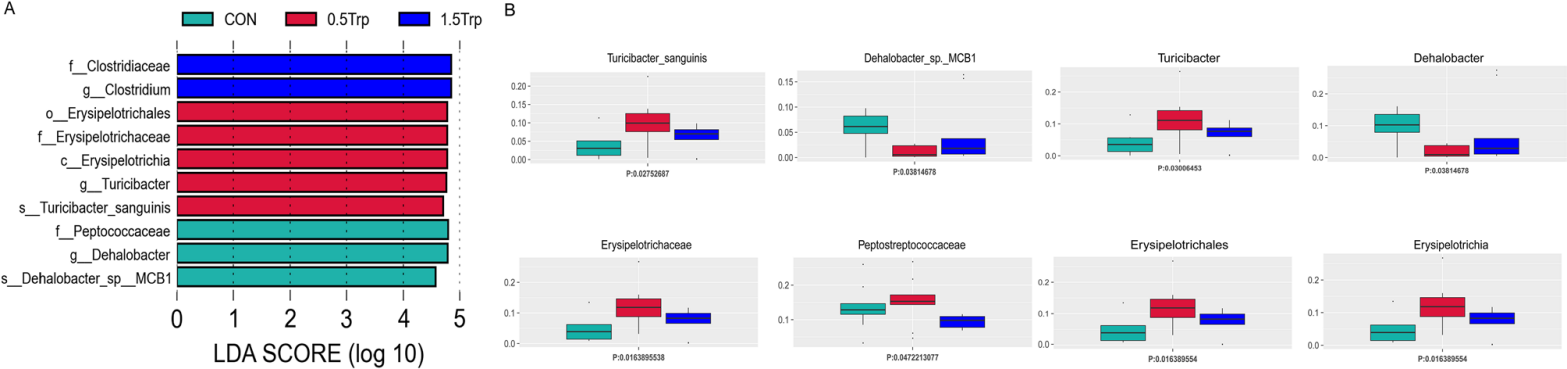
Linear discriminant analysis histogram (**A**) The ordinate shows the taxonomic groups with significant differences among the groups, and the abscissa shows the logarithmic LDA score of each taxonomic group. (**B**) Box plots illustrating the relative abundance of the key discriminant taxa identified in (**A**) across the CON, 0.5Trp, and 1.5Trp groups

Bacterial taxa associated with the CON and Trp-supplemented groups were identified through LEfSe (Linear Discriminant Analysis Effect Size) analysis with a linear discriminant analysis (LDA) score > 4.5 to identify core discriminative taxa across groups. As shown in Fig. 3, taxonomic differences across six classification levels (phylum, class, order, family, genus, and species) were visualized using LDA histograms and assessed with the Kruskal‑Wallis test. In the CON group, *Peptococcaceae*, *Dehalobacter*, and *Dehalobacter*_sp._MCB1 were identified as biomarkers. In the 1.5Trp group, although *Clostridiaceae* and *Clostridium* exhibited LDA scores > 4.5, they did not show statistically significant differences in the Kruskal-Wallis test.

**Fig. 4:**
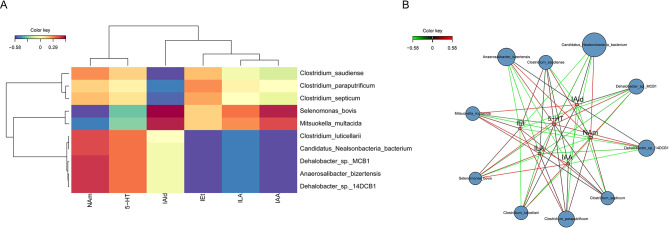
Correlation between gut microbes and metabolites (**A**) Correlation heatmap: rows represent differentially abundant microbial species, columns represent different metabolites, and the color gradient reflects the strength of correlation. (**B**) Green edges indicate negative correlations, red edges indicate positive correlations

To investigate the interactions between the gut microbiota and Trp metabolism, this study employed the mixOmics approach to conduct an integrative correlation analysis of differentially abundant microorganisms at the species level and Trp-related metabolites. As shown in Fig. 4, the abundance of microorganisms such as *Clostridium*_*luticellarii*, *Candidatus*_*Nealsonbacteria*_*bacterium*, *Dehalobacter*_sp._MCB1, *Anaerosalibacter*_*bizertensis* and *Dehalobacter*_sp._14DCB1 showed a negative correlation with the levels of IAA and IEt, but a positive correlation with the levels of NAm and 5-HT. In contrast, *Selenomonas*_*bovis* and *Mitsuokella*_*multacida* exhibited a positive correlation with IAA and IAld, while showing a negative correlation with NAm. Furthermore, the abundance of *Clostridium*_*paraputrificum*, *Clostridium*_*saudiense* and *Clostridium*_*septicum* was negatively correlated with IAld.

### Tryptophan regulates intestinal health by improving intestinal immune status

**Fig. 5:**
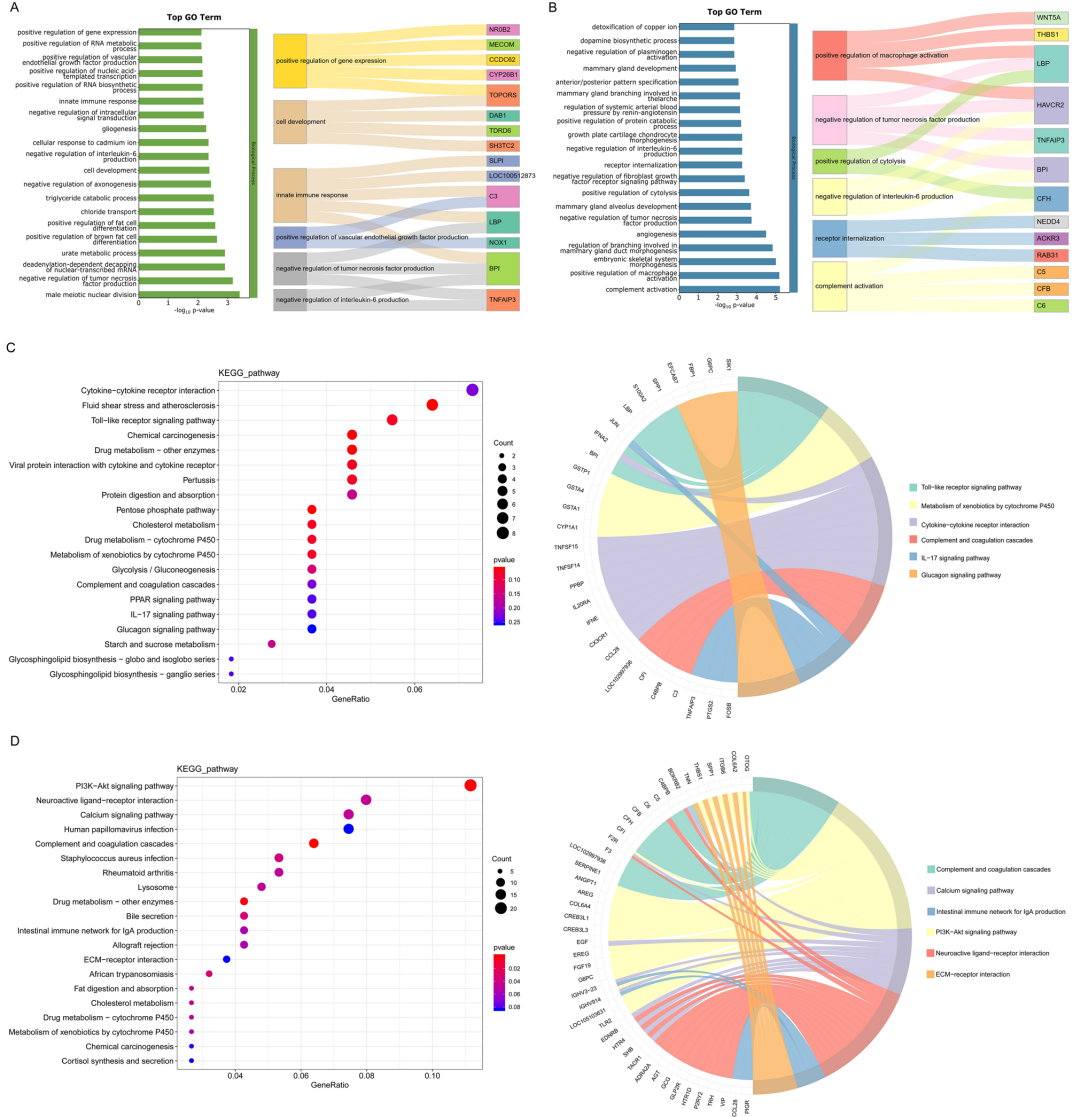
GO and KEGG enrichment analysis of DEGs (**A**) GO biological process enrichment of DEGs in the CON vs. 0.5Trp comparison. (**B**) GO biological process enrichment of DEGs in the CON vs. 1.5Trp comparison. (**C**) Bubble plot and circular network plot of KEGG enrichment analysis of DEGs in the CON vs. 0.5Trp comparison. (**D**) Bubble plot and circular network plot of KEGG enrichment analysis of DEGs in the CON vs. 1.5Trp comparison

To explore potential mechanisms through which Trp affects ileal health, we performed RNA-sequence to quantify ileal gene expression profiles. DEGs were subjected to GO enrichment analysis. The analysis revealed significant enrichment of DEGs in inflammatory and immune-related biological processes. Compared to the CON group, the main immune-related enrichments in the 0.5Trp group were in negative regulation of tumor necrosis factor production, negative regulation of interleukin-6 production, innate immune response, positive regulation of nucleic acid-templated transcription, positive regulation of gene expression, and other pathways (Fig. 5A). The 1.5Trp group was mainly enriched in pathways including complement activation, positive regulation of macrophage activation, negative regulation of tumor necrosis factor production, positive regulation of cytolysis, receptor internalization, and negative regulation of interleukin-6 production (Fig. 5B).

To elucidate the biological mechanisms underlying Trp’s effects on piglet ileal physiology, Kyoto Encyclopedia of Genes and Genomes (KEGG) pathway enrichment analysis revealed significant enrichment of immune and metabolic processes in the 0.5Trp versus CON comparison (Fig. 5C, D). Key enriched pathways included cytokine-cytokine receptor interaction, Toll-like receptor signaling pathway, PPAR signaling pathway, IL-17 signaling pathway, glycolysis/gluconeogenesis, starch and sucrose metabolism, protein digestion and absorption. Among these pathways, DEGs such as secreted phosphoprotein 1 (*SPP1*), S100 calcium binding protein A2 (*S100A2*), lipopolysaccharide-binding protein (*LBP*), interleukin 20 receptor subunit alpha (*IL20RA*), interferon alpha 2 (*IFNA2*), chemokine (C-X3-C Motif) receptor 1 (*CX3CR1*), C-C motif chemokine ligand 28 (*CCL28*) and bactericidal permeability increasing protein (*BPI*) were significantly enriched in these pathways (Fig. 5C). With increasing Trp concentration, KEGG pathways significantly enriched by differential genes were mainly concentrated in immune-related pathways such as PI3K-Akt signaling pathway, Neuroactive ligand-receptor interaction, Complement and coagulation cascades, ECM-receptor interaction and other pathways (Fig.5D).

**Fig. 6:**
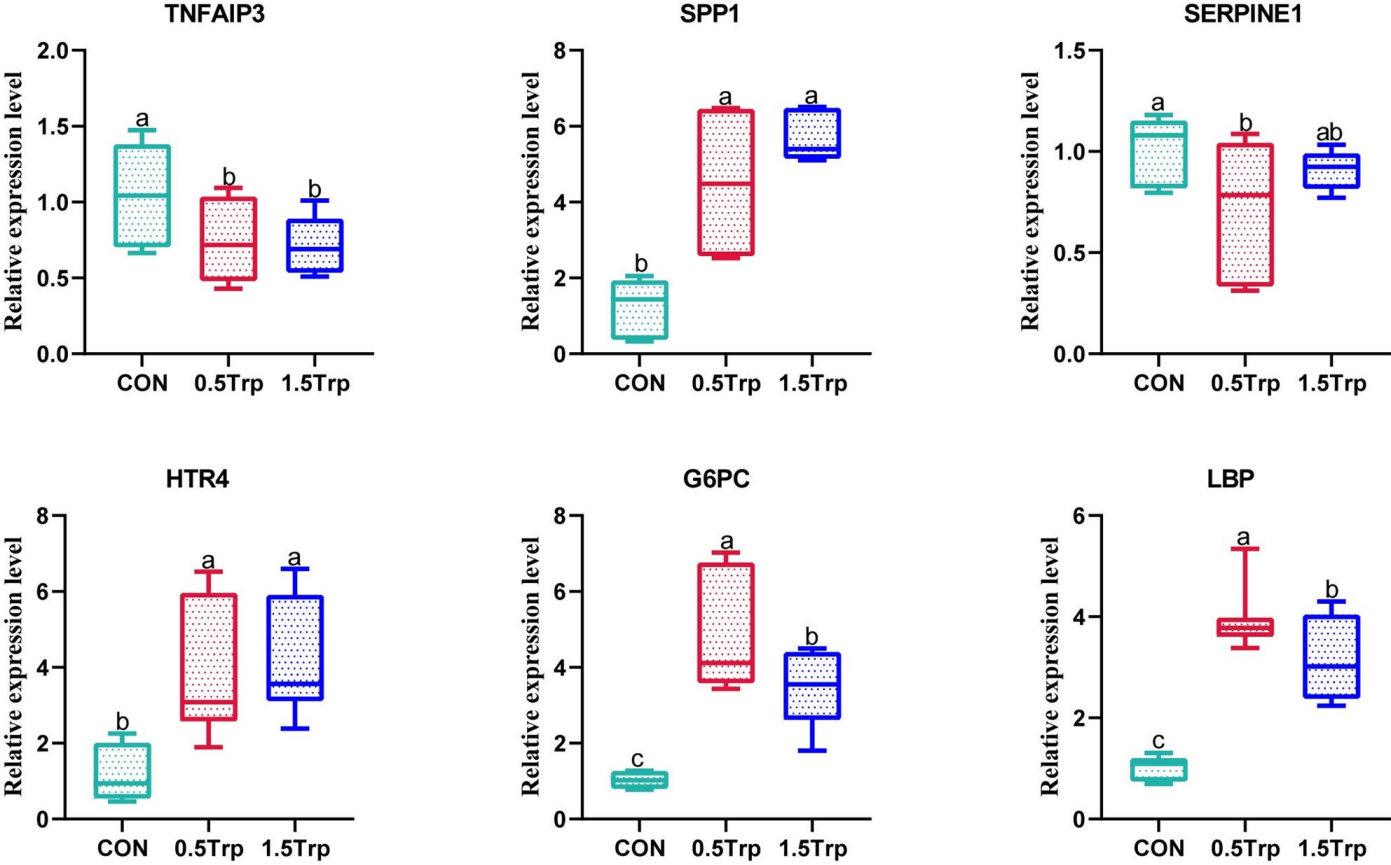
Verification of DEGs Different letters represent significant differences in results (*P* < 0.05)

*LBP*, TNF alpha induced protein 3 (*TNFAIP3*), glucose-6-phosphatase (*G6PC*), *SPP1*, serpin family E member 1 (*SERPINE1*) and 5-hydroxytryptamine receptor 4 (*HTR4*) in the DEGs were selected for verification by RT-qPCR. The experimental results exhibited consistency with the transcriptomic sequencing data, thereby confirming the accuracy of the sequencing methodology (Fig. 6).

**Fig. 7:**
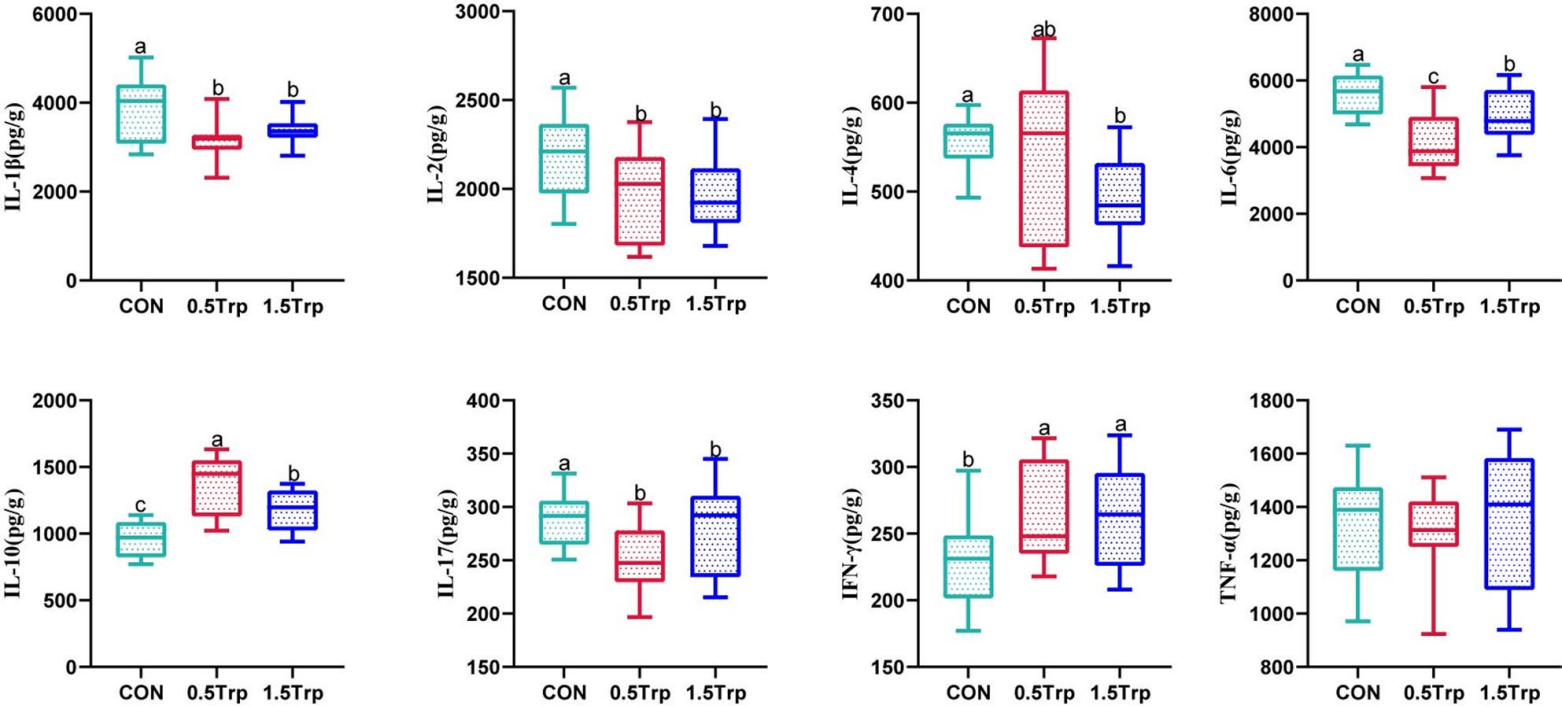
The effect of tryptophan on the immune function of the ileum Different letters represent significant differences in results (*P* < 0.05)

Transcriptomics analysis revealed that Trp significantly modulated the immune status of the piglet ileum. To further investigate whether Trp could regulate the immune status, we quantified ileal cytokine levels using ELISA. Compared with the CON group, the 0.5Trp and 1.5Trp groups showed significantly lower levels of IL-1β, IL-2, IL-4, IL-6, and IL-17, while IL-10 and IFN-γ levels were elevated (*P* < 0.05) (Fig. 7).

### Correlations among DEGs, gut microbiota, and cytokine

**Fig. 8:**
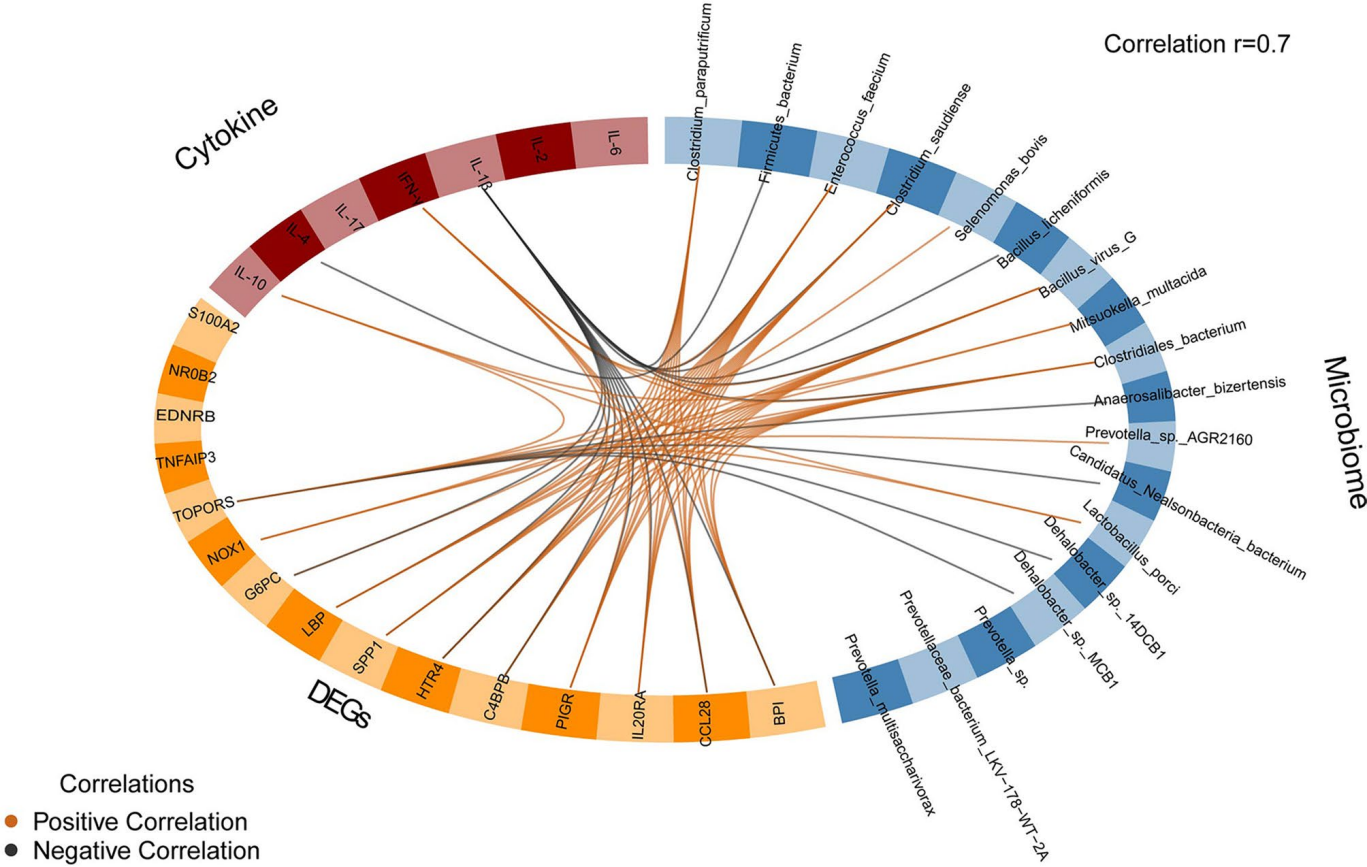
Correlation between multi-omics and cytokines

By integrating metagenomic, transcriptomic, and phenotypic data through DIABLO analysis, we identified association networks between key immune factors and specific microbe - host gene pairs. The results showed that the pro-inflammatory factor IL-1β was negatively correlated with *Clostridium*_*paraputrificum*, *Firmicutes*_*bacterium*, *Enterococcus*_*faecium*, *Clostridium*_*saudiense*, *Bacillus*_*licheniformis*, *Clostridiales*_*bacterium* as well as with the expression of *G6PC*, *LBP*, *SPP1*, *HTR4*, complement component 4 binding protein beta (*C4BPB*), polymeric immunoglobulin receptor (*PIGR*), *IL20RA*, *CCL28*, and *BPI*. In contrast, IFN‑γ exhibited positive correlations with *Enterococcus*_*faecium*, *Bacillus*_*virus*_*G*, *Clostridiales*_*bacterium* and with the expression of *HTR4*, *PIGR*, *IL20RA*, and *CCL28*. Notably, *HTR4*, *PIGR*, *IL20RA*, and *CCL28* displayed opposite association directions in the IL‑1β and IFN‑γ networks, suggesting their context‑dependent dual regulatory functions in immune responses. Additionally, IL‑4 was negatively correlated with *Firmicutes*_*bacterium*, whereas the anti‑inflammatory cytokine IL‑10 showed positive correlations with *Mitsuokella*_*multacida*, *Lactobacillus*_*porci*, and the TOP1-binding arginine/serine-rich protein (*TOPORS*) gene (Fig. 8).

## Discussion

Diarrhea in piglets induces multifaceted alterations in intestinal morphology, physiological function, microbial composition, and immune responses, which collectively compromise gut health [21]. The ileum, serving as both a nutrient absorption hub and a key component of gut-associated lymphoid tissue, plays dual roles in digestion and immune surveillance. Our investigation demonstrated that dietary supplementation with two distinct concentrations of Trp significantly reduced the diarrhea index, with the 1.5Trp group additionally exhibiting increased ADFI. However, the effects of Trp supplementation on growth performance in weaned piglets remain controversial. Previous studies have reported that diets containing adequate Trp levels (0.21%, 0.28%, or 0.35%) significantly improved ADFI, ADG, feed conversion ratio, and reduced diarrhea incidence [22]. Conversely, other investigations observed no discernible impact on growth performance with dietary supplementation of 0.15% or 0.75% Trp [23]. Notably, villus height and crypt depth serve as critical biomarkers for intestinal health [24]. Although Trp supplementation did not significantly affect overall growth performance in this study, piglets in the Trp-treated group exhibited markedly elongated intestinal villi compared to controls. These observations align with earlier reports by Engelsmann [25], suggesting that diarrhea-induced intestinal structural disruption in piglets may be ameliorated through Trp-mediated restoration of intestinal integrity.

Dysbiosis of the gut microbiota is recognized as a primary contributor to diarrhea in weaned piglets [3, 26]. A complex and diverse microbial community is essential for maintaining innate and adaptive immunity as well as gut health in weaned piglets [27, 28]. While the effect of different Trp concentrations on ileal microbiota composition had not been systematically investigated, our metagenomic analysis revealed that Trp supplementation induces significant modulations in ileal microbial diversity, most notably at the phylum level, characterized by increased *Deferribacteres* abundance and decreased *Tenericutes* populations. Previous studies have demonstrated that *Deferribacteres* protect mice from *Salmonella enterica* infection by interfering with pathogen invasion and virulence factor expression [29], while elevated *Tenericutes* levels correlate with pro-inflammatory states [30]. In high-fat diet-induced obese mice, changes in the Trp biosynthesis pathway are related to changes in *Deferribacteres* abundance [31]. In contrast, excessive zinc intake disrupts the microbial balance of piglets and significantly increases the abundance of *Tenericutes*, while hesperidin supplementation with antioxidant properties can significantly reverse this situation [32].

Taxonomic analysis at the genus level and species level revealed four critical shifts: increased *Turicibacter*,* Clostridiales*_*bacterium* and *Turicibacter*_*sanguinis* abundance accompanied by decreased *Chryseobacterium* abundance. Notably, *Turicibacter* demonstrates dual regulatory roles in intestinal immunity and host metabolism, evidenced not only by its significant positive correlation with Trp metabolism [33] but also by its capacity to mitigate *Salmonella*-induced inflammatory susceptibility in B4galnt2-deficient murine models [34] and to enhance porcine growth performance [35]. Importantly, *Turicibacter*_*sanguinis* within this genus demonstrates a unique capacity to modulate Trp catabolism, balancing serotonin synthesis while suppressing KYN production-a critical mechanism for maintaining intestinal barrier homeostasis [36]. Similarly, *Clostridiales_bacterium* exhibits cellulolytic activity and is correlated with enhanced total antioxidant capacity in piglets [37, 38], indicating its potential to improve fiber digestibility and redox homeostasis. Conversely, *Chryseobacterium*, a pathogen associated with infant infections, was significantly enriched in the ileum of *Salmonella*-infected pigs [39, 40]. Collectively, these results indicate that Trp effectively modulates the ileal microbiota, increasing microbial diversity and rebalancing the beneficial-to-pathogenic ratio to promote intestinal health in piglets, as further supported by LEfSe analysis.

Trp, an essential AA obtained through dietary intake in mammals, undergoes microbial metabolism in the gut to produce indoles and their derivatives These bioactive compounds are known to regulate intestinal immune pathways [41, 42]. The present analysis revealed that certain microbial species, such as *Clostridium*_*luticellarii*, were positively correlated with NAm and 5‑HT, suggesting that these microbes may predominantly facilitate the conversion of Trp into NAm and 5‑HT, thereby contributing to host energy metabolism and neuro‑immune regulation [43, 44]. On the other hand, *Selenomonas*_*bovis* and related species showed significant positive associations with indole-derived metabolites (e.g., IAA and IAld), indicating a metabolic preference toward the indole pathway. These indole-based metabolites can act as endogenous ligands of the AHR, playing a role in maintaining intestinal barrier integrity and immune homeostasis [45].

To delineate the mechanisms underlying Trp-mediated ileal health regulation in piglets, we conducted transcriptomic profiling. Notably, most Trp-induced DEGs were enriched in ileal immunity-related pathways, including negative regulation of tumor necrosis factor production, negative regulation of IL-6 production, cytokine-cytokine receptor interaction, Toll-like receptor signaling pathway, IL-17 signaling pathway and PI3K-Akt signaling pathway and other pathways. Through systematic screening, we identified key immune-related genes such as *LBP*, *BPI*, *TNFAIP3* et al. *LBP*, a plasma protein critical for innate immune responses during inflammatory and infection-related diseases [46], has been specifically implicated in *Clostridium perfringens* type C-induced diarrheal pathogenesis. Huang et al. demonstrated that *LBP* overexpression confers protection against bacterial infection-triggered diarrhea in piglets [47]. Similarly, *BPI* participates in extracellular antimicrobial defenses and modulates inflammatory responses during host-pathogen interactions [48]. Importantly, cellular overexpression of *BPI* enhances bactericidal capacity, suggesting its pivotal role in mitigating weaning-associated stress and intestinal immune dysregulation [49].

Numerous studies have demonstrated that AA metabolism critically modulates host physiology, including growth, reproduction, and immune regulation [50]. To further elucidate the impact of Trp on ileal immune function, we systematically investigated cytokine profile alterations through comprehensive biochemical analyses. Diarrheic piglets exhibited elevated pro-inflammatory cytokines, which detrimentally impacted intestinal health. Trp supplementation significantly reduced levels of IL-1β, IL-2, IL-6, and IL-17 while increasing IL-10 and IFN-γ concentrations. Although IL-10 typically suppresses IFN-γ production [12, 51], their concurrent upregulation in Trp-treated piglets suggests nuanced immunomodulatory mechanisms, potentially reflecting complex interplay between regulatory and effector immune signaling in mucosal tissues. As a key member of the interleukin family, IL-6 plays a pivotal role in promoting inflammation [52]. Notably, serum IL-6 levels are significantly elevated in patients with major depressive disorder and demonstrate clinical associations with Trp metabolism [53].

Previous studies have demonstrated that dietary Trp supplementation elevates levels of both the anti‑inflammatory cytokine IL‑10 and the pro‑inflammatory cytokine IFN‑γ [12], consistent with our present observations. IFN-γ, a principal effector cytokine of the immune system, is strictly expressed by lymphoid lineage cells. In this study, we observed a significant positive correlation between IFN-γ and *Enterococcus faecium*. Previous research has indicated that *Enterococcus faecium* can promote the secretion of IFN-γ by IFN-γ⁺ CD8⁺ T cells, and this cytokine, in combination with sorafenib, can induce ferroptosis in hepatocellular carcinoma cells [54]. Furthermore, *Enterococcus faecium* has been shown to reduce inflammatory cytokine production by inhibiting the NF-κB/NLRP3/IL-1β signaling pathway, a mechanism that aligns with our finding of a negative correlation between IL‑1β and *Enterococcus faecium* [55]. At the level of mucosal immunoregulation, the trans-epithelial transport of immunoglobulin A mediated by the polymeric immunoglobulin receptor (*PIGR*) is directly regulated by IFN-γ [56, 57]. The positive correlation between *PIGR* and IFN‑γ observed here supports this regulatory relationship. Notably, the mucosal chemokine *CCL28*, which mediates lymphocyte chemotaxis during chronic *Salmonella* infection [58], was also positively correlated with IFN-γ in our investigation. This finding suggests that *CCL28* and IFN-γ may cooperate in modulating mucosal immune networks. However, the direct regulatory interaction between *CCL28* and IFN-γ, along with the underlying molecular mechanisms, requires further elucidation through functional studies such as gene editing and signaling pathway blockade.

The anti-inflammatory cytokine IL-10, mainly secreted by T cells, B cells, and monocytes, serves as an important mediator in maintaining gut microbiota homeostasis and mucosal immune balance [59]. Our data revealed significant positive correlations between IL-10 and *TOPORS* as well as *Lactobacillus porci*. As a key component of the intestinal commensal microbiota, *Lactobacillus* species have been shown to enhance epithelial barrier function and alleviate intestinal inflammation [60]. For instance, *Lactobacillus helveticus* R0052 has been reported to suppress the expression of pro-inflammatory cytokines while increasing IL-10 levels in a colitis model [61]. In contrast, overexpression of *TNFAIP3* in intestinal epithelial cells exacerbates tumor necrosis factor-induced epithelial damage by activating the Ripoptosome/RIPK1 pathway, promoting cell apoptosis and colitis progression [62]. In IL-10-deficient mice, upregulation of *TNFAIP3* similarly leads to severe colitis, further confirming its pro-inflammatory role [63]. Notably, in the present study, Trp supplementation significantly elevated IL-10 levels while reducing *TNFAIP3* expression, suggesting that Trp may contribute to the maintenance of ileal immune homeostasis by modulating this inflammatory regulatory axis.

It should be noted that this study utilized Bama miniature pigs, a model with a consistent genetic background, to minimize individual variation and focus on elucidating the core mechanisms by which Trp regulates intestinal health. Bama miniature pigs differ from modern commercial pig breeds in growth rate and nutritional requirements. Consequently, the primary value of our conclusions lies in revealing a potential Trp-driven “microbiota-immune axis” regulatory mechanism. This finding provides an important theoretical framework for conducting targeted dose-response validation and applied research in commercial pig breeds. Extrapolation of these findings to commercial pig production will require further dose-response studies tailored to the specific physiological and nutritional characteristics of the target breeds.

## Conclusion

This study demonstrates that dietary tryptophan alleviates diarrhea and enhances intestinal health in weaned piglets by reshaping specific microbial communities and modulating the expression of immune-related genes, leading to a balanced cytokine profile (reduced IL-1β/IL-6, elevated IL-10) and improved systemic homeostasis. The findings, obtained from the genetically homogeneous Bama miniature pig model, reveal a Trp-driven “microbiota‑immune” regulatory framework. As Bama pigs differ from commercial breeds in growth and metabolism, translating these mechanistic insights into practical applications will require further dose‑response validation in target commercial lines.

## Data Availability

The metagenomic sequencing data of ileal contents and ileal transcriptomic data have been deposited in the NCBI Sequence Read Archive database under accession numbers (PRJNA1256929 and PRJNA1257051). The metabolome data reported in this study have been deposited in the OMIX database China National Center for Bioinformation / Beijing Institute of Genomics, Chinese Academy of Sciences (https://ngdc.cncb.ac.cn/omix: accession No. OMIX012051).

## Abbreviations

AA: Amino acid
ADFI: Average daily feed intake
ADG: Average daily gain
AHR: Aryl hydrocarbon receptor
BPI: Bactericidal permeability increasing protein
CCL28: C-C motif chemokine ligand 28
CD: Crypt depth
CX3CR1: Chemokine (C-X3-C motif) receptor 1
DEGs: Differentially expressed genes
ELISA: Enzyme-linked immunosorbent assays
F/G: Feed-to-weight ratio
G6PC: Glucose-6-phosphatase
GO: Gene ontology
HTR4: 5-hydroxytryptamine receptor 4
IAld: Indole-3-carboxaldehyde
IAA: Indole-3-acetic acid
IEt: Tryptophol
IEC: Intestinal epithelial cell
IFNA2: Interferon alpha 2
IFN: Interferon
ILA: Indole-3-lactic acid
IL20RA: Interleukin 20 receptor subunit alpha
IPA: Indole-3-propionic acid
KEGG: Kyoto Encyclopedia of Genes and Genomes
KYN: Kynurenine
KYNA: Kynurenic acid
LBP: Lipopolysaccharide-binding protein
LDA: Linear discriminant analysis
LefSe: Linear discriminant analysis effect size
NAm: Nicotinamide
NF-κB: Nuclear factor κB
ORFs: Open reading frames
RT-qPCR: Real-time quantitative PCR
S100A2: S100 calcium binding protein A2
SERPINE1: Serpin family E member 1
SPP1: Secreted phosphoprotein 1
TOPORS: TOP1 binding arginine/serine rich protein
TNFAIP3: TNF alpha induced protein 3
TNF: Tumor necrosis factor
TRM: Tryptamine
Trp: Tryptophan
VH: Villus height
5-HT: Seronine
